# Evaluation of the novel multi-points surface thermometry cryoballoon in the treatment of paroxysmal atrial fibrillation

**DOI:** 10.3389/fcvm.2025.1703472

**Published:** 2025-12-15

**Authors:** Lu Bai, Ruikun Jia, Xiaochi Sun, Juan Chan, Xinmeng Wang, Shijie Wang, Ming Liu, Yue Shen, Jiayu Sun, Kaijun Cui

**Affiliations:** 1 Department of Cardiology, West China Hospital, Sichuan University, Chengdu, Sichuan, China; 2 Department of Equipment and Material, West China Hospital, Sichuan University, Chengdu, Sichuan, China; 3 Department of Radiology, West China Hospital, Sichuan University, Chengdu, Sichuan, China

**Keywords:** IceMagicTM Cardiac CryoAblation System, cryoballoon ablation, atrial fibrillation, contrast agent, radiation exposure, pulmonary vein isolation (PVI), pulsed-field ablation (PFA)

## Abstract

**Background:**

Cryoballoon ablation (CBA) is extensively utilized to treat paroxysmal atrial fibrillation (PAF). The IceMagicTM Cardiac CryoAblation System (CryoMST, MicroPort EP MedTech CO., Ltd, Shanghai, China) has recently been introduced.

**Objectives:**

To evaluate the clinical efficiency and potential benefits of CryoMST in PAF.

**Methods:**

A propensity-score matched study compared 16 patients with PAF undergoing initial CBA with CryoMST against cohorts treated with Arctic Front Advance™ (*n* = 48), radiofrequency ablation (RFA, *n* = 48), and pulsed-field ablation (PFA, *n* = 16). Peri-procedural and 12-month outcome data were compared among the four groups to assess the efficacy and safety of the CryoMST.

**Results:**

The CryoMST cohort showed significant reductions in total procedure time [83.00 (Q1, Q3: 75.00, 89.25) vs. 102.00 (Q1, Q3: 85.00, 127.75) min], ablation time [20.00 (Q1, Q3: 18.74, 21.00) vs. 46.00 (Q1, Q3: 38.00, 55.75) min], fluoroscopy time [12.93 (Q1, Q3: 11.15, 16.96) vs. 17.85 (Q1, Q3: 12.35, 23.38) min], fluoroscopy dose [81.80 (Q1, Q3: 69.93, 96.03) vs. 200.00 (Q1, Q3: 134.75, 311.50) mGy], and contrast agent volume [16.00 (Q1, Q3: 11.00, 22.00) vs. 52.50 (Q1, Q3: 45.00, 57.00) mL] compared to the Arctic Front Advance™ group (*P* < 0.05). CryoMST accurately predicted pulmonary vein occlusion, showing high concordance with pulmonary vein angiograph (sensitivity 89.3%, specificity 100.0%, Kappa value = 0.87, *p* < 0.001). At 12-month follow-up, rates of arrhythmia recurrence and complications did not differ among the groups.

**Conclusions:**

The CryoMST system demonstrates excellent diagnostic accuracy for pulmonary vein occlusion, obviating the need for routine venography. It significantly reduces procedure time, radiation exposure, and contrast use compared to conventional CBA, while maintaining comparable 12-month efficacy and safety to established ablation modalities for PAF.

## 1 Introduction

**Figure 1:**
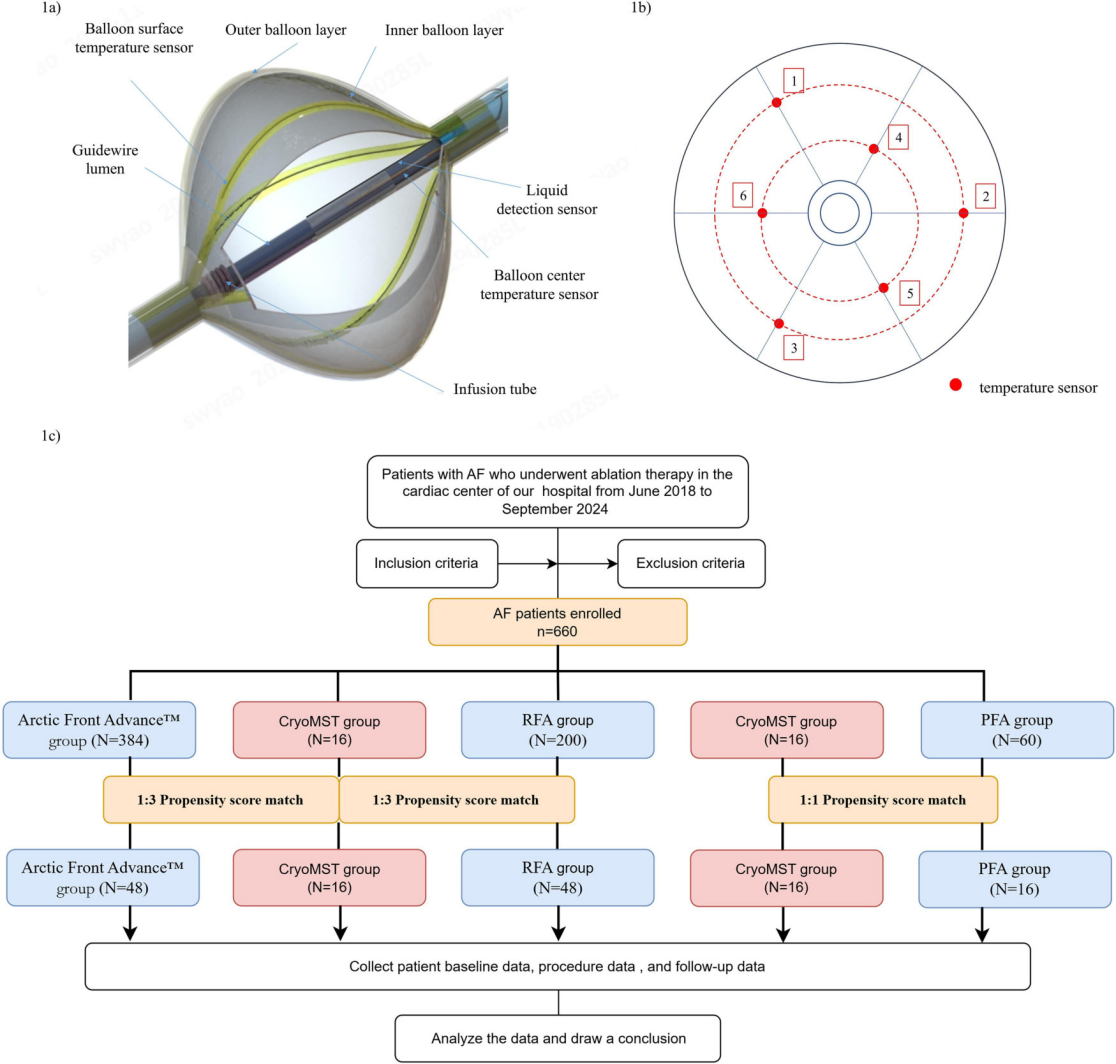
Schematic of the CryoMST and flowchart of this study. **(a)** CryoMST. **(b)** Distribution of the six temperature sensors on the CryoMST. **(c)** Flowchart of this study.

Catheter ablation is a well-established therapy for atrial fibrillation, with cryoballoon ablation (CBA) representing a primary modality (1, 2). However, conventional cryoablation techniques are limited by their dependence on fluoroscopy and contrast media, and by inadequate methods for real-time assessment of pulmonary vein occlusion (3–5). To address these limitations, the novel IceMagicTM Cardiac CryoAblation System (CryoMST, MicroPort EP MedTech CO., Ltd, Shanghai, China) was developed (Figure 1). This system obviates the need for traditional contrast media. Instead, it utilizes the injection of room-temperature saline to verify pulmonary vein occlusion. Integrated surface temperature sensors simultaneously monitor the temperature at the balloon tip, which reflects the temperature at the pulmonary vein blood pool and the balloon-tissue interface. This temperature monitoring provides real-time feedback on both the quality of pulmonary vein occlusion and the progression of tissue freezing, thereby assessing ablation efficacy. Therefore, this study aimed to evaluate the clinical performance and unique advantages of the CryoMST by comparing it against the Arctic Front Advance™ cryoballoon, pulsed-field ablation (PFA), and radiofrequency ablation (RFA) in patients with paroxysmal atrial fibrillation (PAF).

## 2 Methods

### 2.1 Study population

This propensity score-matched comparative study was conducted at West China Hospital of Sichuan University and was approved by the Ethics Committee of the West China Hospital of Sichuan University (Approval No. 2024-224). The study enrolled patients with symptomatic, drug-resistant paroxysmal atrial fibrillation undergoing their first ablation with CryoMST between June and September 2024. The control group comprised a historical cohort of patients with PAF who underwent their first ablation with Arctic Front Advance™, PFA, or RFA at the same centre between June 2018 and September 2024. The inclusion criteria included: (1) undergoing initial ablation therapy for PAF; (2) age between 18 and 85 years; (3) at least one pre-procedure 12-lead Electrocardiogram (ECG) or 24-h ambulatory ECG confirming PAF (according to the European Society of Cardiology (ESC) definition: an episode lasting ≤7 days that terminates spontaneously or within 7 days post-episode); (4) meeting the ESC-recommended indications for catheter ablation procedures; (5) providing written informed consent before the study. Exclusion criteria included: (1) presence of atrial or left atrial appendage thrombosis; (2) left atrial internal diameter >50 mm; (3) valvular heart disease; (4) New York Heart Association (NYHA) class III/IV cardiac function or EF <0.4; (5) uncontrolled hyperthyroidism; (6) contraindications to anticoagulation; (7) prior ablation history; (8) unsuitable pulmonary venous anatomy. The study flow and patient selection process are shown in Figure 1c.

### 2.2 Propensity-score matching

To reduce confounding biases and ensure comparable baselines, R software (version 4.41) was used for propensity score matching with the “nearest” method. A 1:3 matching ratio was applied for the CryoMST group with the Arctic Front Advance™ group and the RFA group, while a 1:1 matching ratio was used for the CryoMST group with the PFA group. The matching was based on the following variables: age, gender, BMI, left atrial size, left ventricular size, left ventricular ejection fraction, hypertension, diabetes, transient ischemic attack (TIA), and CHA2DS2-VASc score, with a caliper value of 0.05.

### 2.3 Preprocedural management

All enrolled patients underwent a standardised preoperative evaluation, which included transthoracic and transesophageal echocardiography, Cardiac computed tomography scans, 12-lead electrocardiogram, and 24-h Holter monitoring, liver and kidney function tests, electrolyte panel, complete blood count, and other relevant examinations. A comprehensive assessment using the CHA2DS2-VASc and HAS-BLED scoring systems was conducted to determine the need for anticoagulant therapy. Antiarrhythmic drugs were discontinued for at least 5 half-lives.

### 2.4 Ablation process

Following successful transseptal puncture access via the femoral vein, a bolus of intravenous heparin was administered, with subsequent doses titrated to maintain an activated clotting time (ACT) of >300 s for cryoballoon procedures and 250–350 s for RFA and PFA procedures.

#### 2.4.1 Cryoballoon ablation

A cryoballoon catheter and a circular mapping catheter were advanced into the left atrium. The ablation sequence for pulmonary veins was standardized: left superior pulmonary vein (LSPV), left inferior pulmonary vein (LIPV), right superior pulmonary vein (RSPV), and right inferior pulmonary vein (RIPV). Pulmonary vein isolation (PVI) was confirmed by demonstrating entrance and exit block using the inner circular mapping catheter after all ablations were completed.

##### 2.4.1.1 CryoMST group

The CryoMST ablation follows standard procedures, with balloon inflation at the pulmonary vein ostium after septal puncture. Room-temperature saline (5 mL) is injected through the guidewire lumen to predict pulmonary vein occlusion by monitoring the blood pool temperature proximal to the balloon. Under normal conditions, the injection of room-temperature saline into the pulmonary vein's blood pool at the balloon's forefront results in a temperature decrease, which is detected by the surface temperature sensors of the CryoMST for a duration of 10–20 s. Poor occlusion allows continuous blood exchange at the occlusion gap between the balloon and the pulmonary vein, causing the temperature of the blood pool, previously lowered by saline, to revert to the temperature of the body fluid quickly. Conversely, effective occlusion prevents blood exchange, maintaining the temperature of the blood pool below body temperature for an extended period.

**Figure 2:**
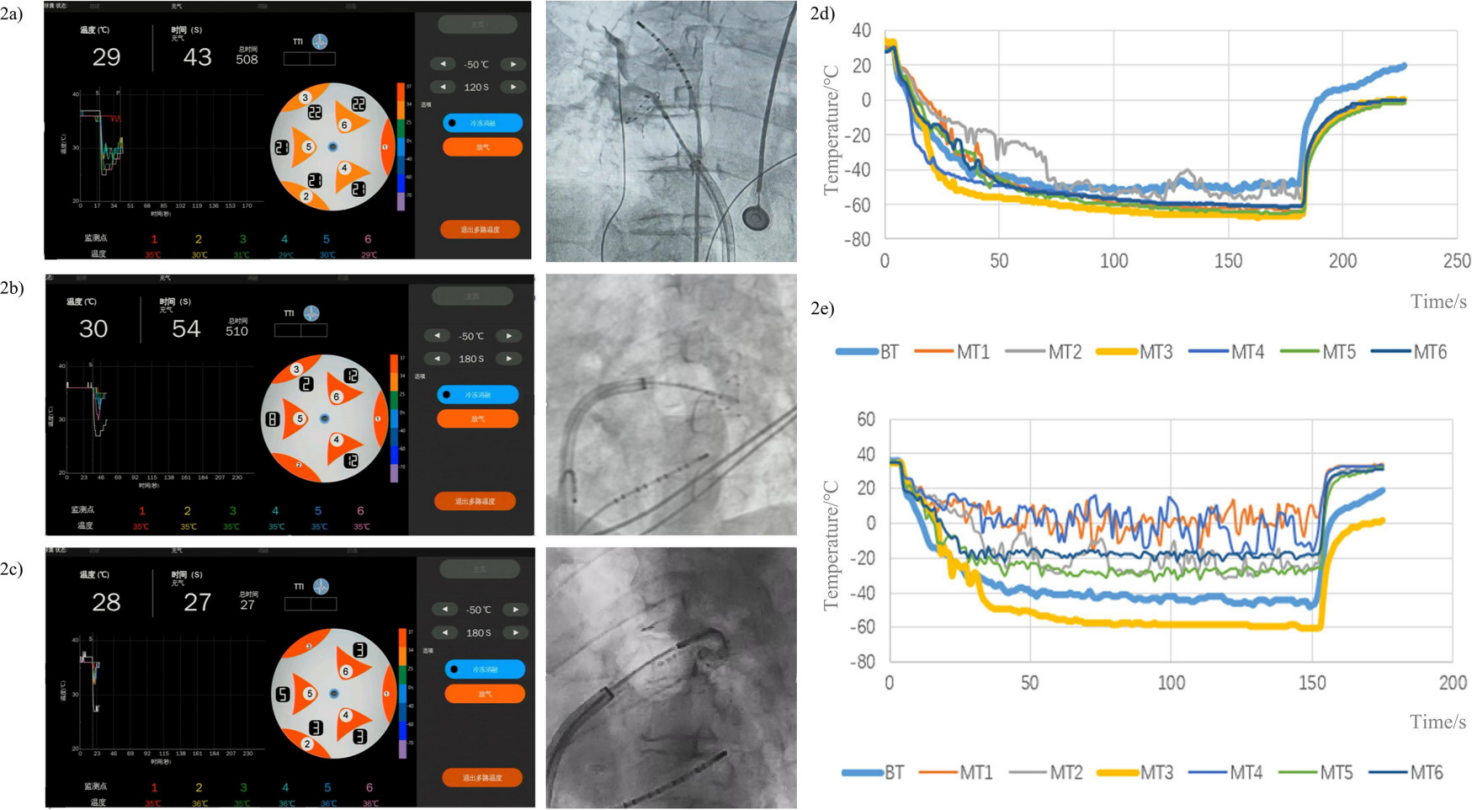
Schematic representation of the CryoMST system predicts pulmonary vein occlusion through the monitoring of balloon surface temperatures. **(a)** Grade I occlusion is indicated by the balloon surface temperature remaining below the central balloon temperature for ≥15 s, with no contrast reflux observed. **(b)** Grade II occlusion occurs when the balloon surface temperature remains below the central balloon temperature for 6–15 s, showing slight contrast reflux and minimal leakage. **(c)** Grade III occlusion involves the balloon surface temperature remaining below the central balloon temperature for less than 6 s, with significant leakage and rapid contrast loss. **(d)** Temperature monitoring curves in the well blocked groups. **(e)** Temperature monitoring curves in the poorly blocked groups. BT: center temperature, MT1-6: surface temperature of six multi-point temperature sensors.

The transient temperature drop, measured by sensors at the balloon tip, was used to grade occlusion. This grading was developed to correlate with conventional angiographic grades: Grade I occlusion is indicated when the balloon surface temperature remains below the central balloon temperature for 15 s or more, with no contrast reflux observed; Grade II occlusion occurs when the balloon surface temperature stays below the central balloon temperature for 6–15 s, showing slight contrast reflux and minimal leakage; Grade III occlusion involves the balloon surface temperature remaining below the central balloon temperature for less than 6 s, with significant leakage and rapid loss of contrast. The balloon position was adjusted until at least a Grade II occlusion was achieved, which was then confirmed with a single confirmatory angiogram. Cryoablation was initiated only after achieving angiographic Grade I or II occlusion (Figure 2).

If time-to-isolation (TTI) was observed within 60 s, the freeze was extended to 180 s, followed by a 120-s bonus application. If TTI was not achieved within 60 s, the application was terminated, and the balloon was repositioned. During right-sided pulmonary vein ablation, continuous phrenic nerve pacing was performed from the superior vena cava, and energy delivery was immediately halted upon detection of diaphragmatic paresis.

##### 2.4.1.2 The arctic front advance™ group

The Arctic Front Advance™ system adheres to the same standard operating procedures. Pulmonary vein occlusion was exclusively assessed by conventional venography. Angiographic occlusion was graded as follows: Grade I occlusion indicates no contrast agent reflux into the atrium; Grade II occlusion indicates mild contrast agent reflux with minimal leakage; Grade III occlusion indicates severe leakage with rapid contrast agent loss. Ablation was performed only after achieving Grade I or II occlusion. The ablation protocol and procedural endpoints were identical to the CryoMST group.

#### 2.4.2 Pulsed-field ablation

Following transseptal puncture, a three-dimensional electroanatomical model was constructed for PVI using biphasic wave parameters at 1800V. Ablation voltage or discharge count was adjusted based on electrical isolation. The procedural endpoint was electrical isolation of all pulmonary veins, confirmed by entrance and exit block 20 min post-ablation.

#### 2.4.3 Radiofrequency ablation

A three-dimensional electroanatomic map was created using a high-density mapping catheter. Point-by-point circumferential PVI was performed using an ablation catheter. The endpoint was the elimination of all pulmonary vein potentials, confirmed by the entrance and exit block.

### 2.5 Post-ablation follow-up and data collection

Outpatient follow-up visits were conducted at 3, 6, 9, and 12 months postoperatively and included a clinical assessment, a 12-lead electrocardiogram (ECG), and 24 h Holter monitoring. Any episodes of AF, atrial flutter, or atrial tachycardia lasting 30 s or more after a three-month blank period were defined as a late recurrence. Baseline, intraoperative, and follow-up data were collected for enrolled patients.

### 2.6 Statistical analysis

Propensity score calculations were conducted using R version 4.41 with the “nearest” matching method. Data processing and analysis were performed using the SPSS 26.0 software package. Continuous variables were expressed as the mean ± standard deviation (*x¯* ± *s*) or the median (Q1, Q3) based on normality. Intergroup comparisons among normally distributed data were performed using *t*-tests or ANOVAs. Non-normally distributed data were compared between groups using the Wilcoxon rank-sum test or Kruskal–Wallis test. Categorical data were expressed as percentages, and the Chi-square (*χ*^2^) test or Fisher exact tests were used for group comparisons. Survival analysis was performed using the Kaplan–Meier method. Sensitivity, specificity, positive predictive value (PPV), negative predictive value (NPV), and their 95% confidence intervals (CIs) were calculated based on the binomial distribution, with pulmonary venography serving as the reference standard. Statistical significance was set at the *p* < 0.05 level.

## 3 Result

### 3.1 Comparison of baseline information

Between June and September 2024, 16 patients with PAF undergoing CryoMST cryoablation at West China Hospital of Sichuan University were enrolled in the study. The control group comprised 644 patients with PAF who underwent Arctic Front Advance™ cryoablation, RFA, or PFA at the same center between June 2018 and September 2024. To control for baseline confounders and ensure group comparability, propensity score matching was applied. This resulted in a final study population of 128 patients: 16 in the CryoMST group, 48 in the Arctic Front Advance™ group, 16 in the PFA group, and 48 in the RFA group. Baseline characteristics were balanced and comparable across these groups (Table 1).

**Table 1:** Baseline characteristics of patients in the CryoMST group, arctic front advance™ group, PFA group, and RFA group.

Characteristic	CryoMST (*n* = 16)	Arctic Front Advance™ (*n* = 48)	PFA (*n* = 16)	RFA (*n* = 48)	*P*
Age (years)	56.50 (47.25, 63.50)	56.00 (49.00, 63.75)	57.00 (52.00, 64.50)	62.50 (52.25, 66.00)	0.068
Male	10 (62.50%)	31 (64.58%)	5 (31.25%)	22 (45.83%)	0.070
BMI (kg/m^2^)	23.93 ± 2.66	23.89 ± 2.57	23.67 ± 3.42	23.91 ± 2.47	0.990
LA diameter (mm)	33.75 ± 4.27	33.90 ± 4.25	34.06 ± 4.85	33.78 ± 5.80	0.998
LV diameter (mm)	46.00 (44.00, 50.00)	47.00 (45.00, 49.75)	45.50 (44.00, 49.75)	47.00 (44.00, 50.00)	0.825
LVEF (%)	66.69 ± 3.98	65.83 ± 6.01	67.56 ± 3.71	67.10 ± 4.97	0.559
Hypertention	6 (37.50%)	22 (45.83%)	8 (50.00%)	22 (45.83%)	0.947
Diabetes	2 (12.50%)	7 (14.58%)	1 (6.25%)	12 (25.00%)	0.342
TIA	0 (0.00%)	0 (0.00%)	0 (0.00%)	0 (0.00%)	1.000
CHA2DS2-VASc	1.00 (0.25, 2.00)	1.00 (0.00, 2.00)	1.00 (0.25, 1.75)	1.00 (1.00, 2.00)	0.585

Data are are presented as mean ± SD or median (IQR). Categorical variables are expressed as a number (percentage). BMI: Body Mass Index, calculated as weight in kilograms divided by the square of height in meters. The CHA2DS2-VASc score assesses the risk of stroke in patients with atrial fibrillation, ranging from 0 to 9, with higher scores indicating a higher risk.

### 3.2 Procedure data

**Figure 3:**
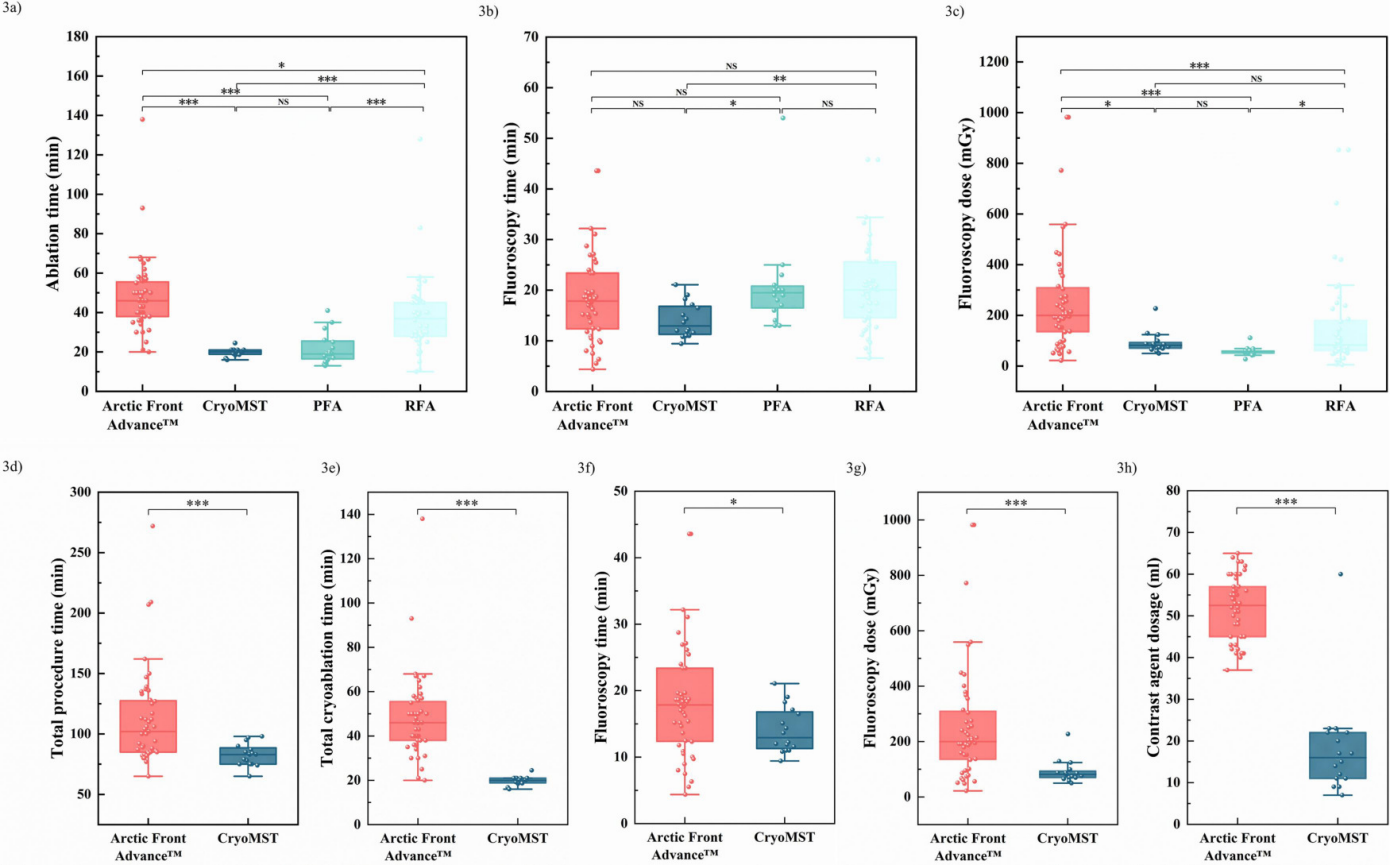
Intraoperative Data Comparisons. Comparison of metrics between different groups includes: **(a)** ablation time, **(b)** fluoroscopy time, and **(c)** fluoroscopy dose among the CryoMST group, Arctic Front Advance™ group, PFA group, and RFA group. Additionally, comparison of **(d)** total procedure time, **(e)** total ablation time, **(f)** fluoroscopy time, **(g)** fluoroscopy dose, and **(h)** contrast agent usage between the CryoMST group and the Arctic Front Advance™ group. ***p < 0.001, **p < 0.01, *p < 0.05.

All patients achieved planned pulmonary vein isolation successfully. The CryoMST group demonstrated significant reductions in total procedure time [83.00 (Q1, Q3: 75.00, 89.25) vs. 102.00 (Q1, Q3: 85.00, 127.75) min], ablation time [20.00 (Q1, Q3: 18.74, 21.00) vs. 46.00 (Q1, Q3: 38.00, 55.75) min], fluoroscopy time [12.93 (Q1, Q3: 11.15, 16.96) vs. 17.85 (Q1, Q3: 12.35, 23.38) min], fluoroscopy dose [81.80 (Q1, Q3: 69.93, 96.03) vs. 200.00 (Q1, Q3: 134.75, 311.50) mGy] and contrast agent volume [16.00 (Q1, Q3: 11.00, 22.00) vs. 52.50 (Q1, Q3: 45.00, 57.00) mL] compared to the Arctic Front Advance™ group (*P* < 0.05). No statistically significant difference was observed in the success rate of acute PVI between these groups. Compared to the RFA group, the CryoMST group had shorter ablation times [20.00 (Q1, Q3: 18.74, 21.00) vs. 37.00 (Q1, Q3: 28.00, 45.00) min], fluoroscopy time [12.93 (Q1, Q3: 11.15, 16.96) vs. 20.05 (Q1, Q3: 14.55, 25.58) min], fluoroscopy dose [81.80 (Q1, Q3: 69.93, 96.03) vs. 83.30 (Q1, Q3: 60.80, 182.30) mGy] (*P* < 0.05). When compared with the PFA group, the CryoMST group showed a slight reduction in fluoroscopy time [12.93 (Q1, Q3: 11.15, 16.96) vs. 19.50 (Q1, Q3: 16.25, 20.90) min, *P* < 0.05]. However, no significant differences were observed in fluoroscopy dose or ablation time (Figure 3, Tables 2, 3). In the CryoMST group, the first 8 patients were compared with the latter 8 patients in terms of the learning curve. As operators gained familiarity with the CryoMST, fluoroscopy time decreased (16.57 ± 3.32 min vs. 11.44 ± 1.18 min, *p* < 0.05) compared to earlier procedures. Fluoroscopy dose and contrast agent usage also showed a reduction, although this did not reach statistical significance (Supplementary Figure 1 and Table 2).

**Table 2:** Ablation procedure parameters of four groups.

Variables	CryoMST (*n* = 16)	Arctic Front Advance™ (*n* = 48)	PFA (*n* = 16)	RFA (*n* = 48)	*P*
Fluoroscopy time (min)	12.93 (11.15, 16.96)dc	17.85 (12.35, 23.38)cbd	19.50 (16.25, 20.90)bac	20.05 (14.55, 25.58)ab	0.005
Fluoroscopy dose (mGy)	81.80 (69.93, 96.03)c	200.00 (134.75, 311.50)a	57.35 (49.23, 59.70)dc	83.30 (60.80, 182.30)bc	<0.001
Ablation time (min)	20.00 (18.74, 21.00)c	46.00 (38.00, 55.75)a	19.00 (16.65, 25.50)dc	37.00 (28.00, 45.00)b	<0.001

Continuous data are presented as median (IQR). Figures use a letter-based significance notation system. Label each set of data in descending order, using the letters a, b, c, and d. If two groups share the same letter, it indicates no significant difference; if they do not share any letter, it indicates a significant difference.

**Table 3:** Procedural parameters for cryoballoon ablation.

Variables	CryoMST (*n* = 16)	Arctic Front Advance™ (*n* = 48)	Diff (95%CI)	*p*
Fluoroscopy time (min)	12.93 (11.15, 16.96)	17.85 (12.35, 23.38)	3.73 (0.08, 6.85)	0.042
Fluoroscopy dose (mGy)	81.80 (69.93, 96.03)	200.00 (134.75, 311.50)	119.70 (71.00, 167.40)	<0.001
Contrast agent dosage (mL)	16.00 (11.00, 22.00)	52.50 (45.00, 57.00)	35.00 (31.00, 40.00)	<0.001
Total procedure time (min)	83.00 (75.00, 89.25)	102.00 (85.00, 127.75)	14.00 (5.00, 29.00)	<0.001
Total cryoablation time (min)	20.00 (18.74, 21.00)	46.00 (38.00, 55.75)	25.50 (19.55, 31.00)	<0.001
Acute pulmonary vein isolation	64/64 (100.00%)	192/192 (100.00%)	0.00% (0.00%, 0.00%)	1.000
LSPV				
Temp at 30 s, °C	−30.94 ± 3.80	−29.17 ± 5.12	−1.77 (−4.56, 1.02)	0.209
Temp at 60 s, °C	−42.50 (−45.00, −41.00)	−40.00 (−42.75, −35.00)	4.00 (1.00, 7.00)	0.003
Minimum freeze temperature, °C	−50.50 (−51.00, −50.00)	−47.00 (−49.00, −42.25)	4.00 (2.00, 6.00)	0.002
Number of cryo applications	2.00 (2.00, 3.00)	2.00 (1.00, 2.00)	−1.00 (−1.00, 0.00)	<0.001
Ablation time, s	300.00 (300.00, 360.00)	300.00 (180.00, 300.00)	−60.00 (−120.00, 0.00)	0.004
TTI, s	35.00 (24.00, 77.00)	43.00 (30.00, 57.00)	6.00 (−8.00, 19.00)	0.269
LIPV				
Temp at 30 s, °C	−29.50 (−32.75, −26.00)	−27.00 (−31.00, −26.00)	2.00 (−1.00, 4.00)	0.161
Temp at 60 s, °C	−39.50 (−41.50, −36.25)	−35.00 (−38.75, −32.25)	4.00 (1.00, 6.00)	0.009
Minimum freeze temperature, °C	−45.38 ± 3.83	−40.94 ± 4.82	−4.44 (−7.09, 1.78)	0.001
Number of cryo applications	2.00 (2.00, 2.00)	1.00 (1.00, 2.00)	−1.00 (−1.00, 0.00)	0.002
Ablation time, s	300.00 (300.00, 300.00)	180.00 (180.00, 300.00)	−120.00 (−120.00, 0.00)	0.003
TTI, s	25.00 (20.00, 36.00)	24.00 (20.00, 40.00)	0.00 (−6.00, 10.00)	0.924
RSPV				
Temp at 30 s, °C	−34.00 (−36.00, −30.25)	−33.50 (−35.00, −29.00)	1.0 (−1.00, 4.00)	0.231
Temp at 60 s, °C	−46.56 ± 2.83	−41.85 ± 4.71	−4.71 (−6.69, −2.73)	<0.001
Minimum freeze temperature, °C	−50.00 (−51.75, −50.00)	−50.00 (−53.00, −47.00)	1.00 (−1.00, 3.00)	0.453
Number of cryo applications	2.00 (2.00, 2.00)	1.00 (1.00, 2.00)	−1.00 (−1.00, 0.00)	0.007
Ablation time, s	300.00 (261.75, 300.00)	180.00 (180.00, 300.00)	−60.00 (−120.00, 0.00)	0.053
TTI, s	30.00 (23.00, 42.00)	33.00 (23.00, 43.00)	2.00 (−8.00, 11.00)	0.634
RIPV				
Temp at 30 s, °C	−31.88 ± 5.89	−29.15 ± 5.59	−2.73 (−6.00, 0.54)	0.100
Temp at 60 s, °C	−41.88 ± 4.91	−36.38 ± 5.82	−5.50 (−8.74, −2.26)	0.001
Minimum freeze temperature, °C	−50.00 (−50.00, −46.50)	−41.00 (−47.75, −38.00)	7.00 (3.00, 10.00)	0.002
Number of cryo applications	2.00 (2.00, 2.00)	2.00 (1.00, 2.00)	0.00 (−1.00, 0.00)	0.173
Ablation time, s	300.00 (231.75, 300.00)	240.00 (180.00, 317.00)	0.00 (−90.00, 17.00)	0.372
TTI, s	27.00 (19.25, 44.00)	33.50 (24.75, 44.00)	5.00 (−7.00, 15.00)	0.285

Data are presented as mean ± SD or median (IQR).

Intraoperative cryoballoon parameters for each pulmonary vein were assessed, with results detailed in Table 3. Notably, the CryoMST group demonstrated lower ablation temperatures in the 30 s and 60 s compared to the Arctic Front Advance™ group in all four pulmonary veins. However, the difference in 30 s was not statistically significant. The lowest ablation temperatures in the CryoMST group were lower than those in the Arctic Front Advance™ group for all pulmonary veins except RSPV (*p* < 0.05). Additionally, the CryoMST group exhibited a higher number of cryo application in all pulmonary veins except RIPV compared to the Arctic Front Advance™ group (*p* < 0. 05). Furthermore, there was no statistically significant difference in TTI of pulmonary veins between these two cryoballoon ablation groups (Table 3).

As the key endpoint of this study, with pulmonary venography as the gold standard, when pulmonary venography indicated grade I occlusion, CryoMST predicted pulmonary vein occlusion with a sensitivity of 89.3% (95% CI: 78.5%–95.0%), specificity of 100.0% (95% CI: 90.6%–100.0%), positive predictive value of 100.0% (95% CI: 92.9%–100.0%), and negative predictive value of 86.0% (95% CI: 72.7%–93.4%). This demonstrated high concordance with pulmonary venography in assessing pulmonary vein occlusion (Kappa = 0.87, 95% CI: 0.77–0.97; *p* < 0.001). For individual pulmonary veins, the sensitivity of CryoMST for predicting occlusion was as follows: LSPV, 100.0% (95% CI: 81.6%–100.0%); LIPV, 83.3% (95% CI: 55.2%–95.3%); RSPV, 85.7% (95% CI: 60.1%–96.0%); and RIPV, 84.6% (95% CI: 57.8%–95.7%). The specificity of CryoMST for predicting occlusion was as follows: LSPV, 100.0% (95% CI: 74.1%–100.0%); LIPV, 100.0% (95% CI: 72.2%–100.0%); RSPV, 100.0% (95% CI: 61.0%–100.0%); and RIPV, 100.0% (95% CI: 72.2%–100.0%). The positive predictive values of CryoMST for predicting occlusion were as follows: LSPV, 100.0% (95% CI: 81.6%–100.0%); LIPV, 100.0% (95% CI: 72.2%–100.0%); RSPV, 100.0% (95% CI: 75.8%–100.0%); and RIPV, 100.0% (95% CI: 74.1%–100.0%). The negative predictive values were 100.0% (95% CI: 74.1%–100.0%) for LSPV, 83.3% (95% CI: 55.2%–95.3%) for LIPV, 75.0% (95% CI: 40.9%–92.9%) for RSPV, and 83.3% (95% CI: 55.2%–95.3%) for RIPV (Table 4).

**Table 4:** Sensitivity, specificity, positive predictive value, negative predictive value, and consistency of the CryoMST for PV occlusion verification.

Variables	Sensitivity (95%CI)	Specificity (95%CI)	Positive Predictive Value (95%CI)	Negative Predictive Value (95%CI)	Kappa (95%CI)	Kappa-P
LIPV	83.3% (55.2%, 95.3%)	100.0% (72.2%, 100.0%)	100.0% (72.2%, 100.0%)	83.3% (55.2%, 95.3%)	0.82 (0.58, 1.00)	<0.001
LSPV	100.0% (81.6%, 100.0%)	100.0% (74.1%, 100.0%)	100.0% (81.6%, 100.0%)	100.0% (74.1%, 100.0%)	1.00 (1.00, 1.00)	<0.001
RSPV	85.7% (60.1%, 96.0%）	100.0% (61.0%, 100.0%)	100.0% (75.8%, 100.0%)	75.0% (40.9%, 92.9%)	0.78 (0.50, 1.00)	<0.001
RIPV	84.6% (57.8%, 95.7%)	100.0% (72.2%, 100.0%)	100.0% (74.1%, 100.0%)	83.3% (55.2%, 95.3%)	0.83 (0.60, 1.00)	<0.001
Total	89.3% (78.5%, 95.0%）	100.0% (90.6%, 100.0%)	100.0% (92.9%, 100.0%)	86.0% (72.7%, 93.4%)	0.87 (0.77, 0.97)	<0.001

### 3.3 Post-procedural follow-up

**Figure 4:**
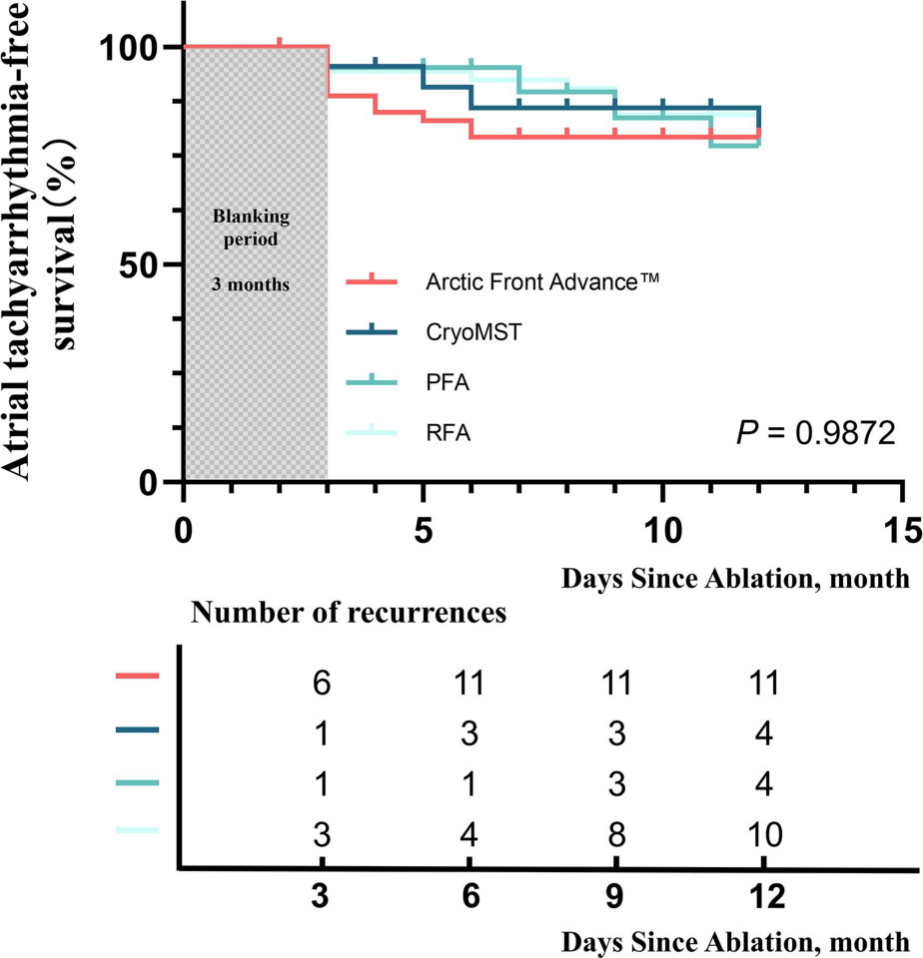
Survival Analysis. Kaplan–Meier survival curves for recurrence of non-atrial rapid arrhythmias across different groups.

At a median follow-up of 12 months, the CryoMST system demonstrated clinical efficacy and safety comparable to established ablation strategies. Kaplan–Meier analysis revealed no statistically significant difference in 1-year recurrence-free survival rates among the CryoMST, Arctic Front Advance™, PFA, and RFA groups (*P* = 0.9872) (Figure 4). No severe complications, including phrenic nerve paralysis, peripheral vascular issues, stroke, death, or other adverse events, were observed in any group during the study period.

## 4 Discussion

This is the first study to our knowledge to directly compare the CryoMST with the second-generation cryoballoon (Arctic Front Advance™), RFA, and PFA for the treatment of PAF. Our principal finding is that the CryoMST system, by integrating a temperature monitoring system for pulmonary vein occlusion assessment, significantly reduces radiation exposure and contrast medium usage compared to conventional cryoablation, while maintaining comparable 12-month safety and efficacy to all three established ablation modalities.

The current gold standard for assessing pulmonary vein occlusion during cryoablation is pulmonary venography, an x-ray fluoroscopic technique that visualizes contrast reflux in the pulmonary veins (3). This dependence creates a clinical dilemma: while necessary for procedural guidance, the requisite use of fluoroscopy and contrast agents introduces significant safety concerns for both patients and operators, such as radiation-associated risks and the potential for contrast-induced acute kidney injury (4, 5). To mitigate the reliance on fluoroscopy and contrast, several alternative strategies have been explored. Echocardiographic guidance, using either transesophageal (TEE) or intracardiac (ICE) probes, has proven effective in reducing radiation, offering real-time anatomical visualization (6–8). However, the widespread adoption of these echocardiographic techniques is hampered by practical limitations. TEE is invasive, often poorly tolerated during prolonged procedures, and requires dedicated personnel who may not be readily available. Meanwhile, the high cost of ICE remains a major barrier to its integration into standard clinical workflow. During cryoablation, abrupt fluctuations in balloon pressure can lead to balloon displacement and incomplete pulmonary vein occlusion. These minor shifts may not be reliably detected under fluoroscopy, as pulmonary vein angiography typically provides only static, initial confirmation of balloon positioning, lacking the capacity for real-time monitoring of its stability throughout the procedure (9). This has spurred interest in dynamic, non-radiologic assessment methods. Saline has been investigated as an alternative to contrast agents during cryoablation procedures. Early animal studies demonstrated the principle of using saline injection and temperature sensing to predict occlusion (10). Further studies explored the feasibility of replacing contrast agents with saline in the KODEX-EPD system (Philips, Netherlands), which detects leaks and assesses pulmonary vein occlusion by measuring dielectric changes caused by differences in electrical conductivity between blood and saline (11, 12). Yu Liao et al. integrated the KODEX-EPD system with ICE to provide real-time feedback for balloon positioning during post-ablation procedures (9). Despite their innovation, these approaches have faced barriers to broad clinical uptake, including steep learning curves, high procedural costs, poor reproducibility, and a lack of standardized, validated protocols.

Our study demonstrates that the CryoMST system provides an effective solution to this longstanding challenge. By integrating a multi-point surface thermometry system, it leverages a simple injection of room-temperature saline to provide real-time, dynamic feedback on occlusion quality. Our study indicates that the CryoMST group experienced significant reductions in fluoroscopy time, radiation dose, and contrast volume compared to the conventional cryoballoon cohort. Remarkably, these procedural benefits persisted even though our study protocol mandated a confirmatory angiogram, suggesting potential for further radiation exposure reduction in future studies. Additionally, the method showed excellent concordance with the angiographic gold standard (Kappa = 0.87, 95% CI: 0.77–0.97; *p* < 0.001), achieving a sensitivity of 89.3% and a perfect specificity of 100.0%. Additionally, the technology showed a favorable learning curve, which is essential for clinical adoption. Nonetheless, it should be acknowledged that operator experience and temporal differences in data collection periods between the control and experimental groups may influence the observed reductions in radiation time, dose, and procedure duration.

PFA has garnered significant attention in atrial fibrillation treatment for its procedural speed and tissue selectivity (13, 14). However, its long-term efficacy is still under investigation, and concerns remain regarding high initial costs and unique complications such as coronary spasm and hemolysis-induced renal injury (15, 16). Our 12-month follow-up data indicate that CryoMST offers comparable clinical efficacy and safety to PFA, RFA, and conventional CBA. This positions CryoMST not merely as an incremental improvement but as a highly competitive therapeutic option, enhancing the safety and efficiency of a well-established ablation modality without the cost barrier or novel safety concerns of other emerging technologies.

Another noteworthy observation is the significant difference in temperature metrics at 30 and 60 s of ablation, as well as the minimum ablation temperature, between the CryoMST and Arctic Front Advance™ groups. Through further clinical application of this device, we have gained deeper insights into its usage. Therefore, regarding the differences in ablation temperatures observed at different time points between the two cryoablation devices, we offer the following explanation and have made corresponding revisions in the Discussion section. The modifications are as follows: This is directly attributable to its novel multi-sensor design. Unlike conventional systems, CryoMST provides real-time temperature data from both the balloon-tissue interface and the distal blood pool. This dual-monitoring capability enables an objective, non-radiographic assessment of pulmonary vein occlusion and biophysical parameters of lesion formation. It empowers the operator to optimize balloon positioning and energy delivery with high precision, leading to more efficient and profound cooling profiles. Given that tissue temperature dynamics are a critical determinant of lesion durability, this direct measurement capability represents a significant technological advance (17, 18). Therefore, a key direction for future research is to investigate whether this enhanced thermal monitoring translates into improved procedural accuracy and long-term clinical outcomes.

## 5 Limitations

This study has several limitations that warrant consideration. First, its single-center, non-randomized design is a primary constraint. Although propensity score matching was employed to balance baseline characteristics across the four groups, the potential for residual confounding variables, unmeasured selection bias, and temporal trends cannot be entirely dismissed. Second, while the duration of the balloon surface temperature remaining below the central lumen temperature shows potential as a predictor for acute pulmonary vein isolation, this criterion requires further statistical validation in larger clinical cohorts before it can be established as a reliable endpoint. Third, due to the recent introduction of the CryoMST system, only short-term outcomes could be assessed and were compared against historical controls. The absence of long-term arrhythmia-free survival data is a significant limitation.

## 6 Conclusion

The study indicates that CryoMST, compared to the Arctic Front Advance™, significantly reduces radiation exposure and reliance on contrast agents by employing real-time surface temperature measurement technology and substituting contrast agents with saline solution. It further demonstrates high predictive accuracy for pulmonary vein occlusion. Moreover, its short-term efficacy is comparable to RFA and PFA. In summary, the technological advancements inherent in CryoMST present a substantial potential for optimizing clinical outcomes in atrial fibrillation ablation. Future rigorous, prospective randomized controlled trials are necessary to comprehensively evaluate the long-term performance and clinical benefits of this system relative to conventional ablation devices.

## Data Availability

The original contributions presented in the study are included in the article/Supplementary Material, further inquiries can be directed to the corresponding author/s.

## Supplementary material

Supplementary Figure 1 Learning Curves of the CryoMST Group.

Supplementary Table 1 Criteria for blockage grades using the CryoMST and corresponding fluoroscopy-guided pulmonary venography blockage grade assessment.

Supplementary Table 2 Comparison of fluoroscopy time, fluoroscopy dose, and contrast agent usage between the first 8 patients and the second 8 patients in the CryoMST group.
