# Supplementary material for: Evaluation of the novel multi-points surface thermometry cryoballoon in the treatment of paroxysmal atrial fibrillation
Source: Front Cardiovasc Med. 2025 Dec 15;12:1703472. doi: 10.3389/fcvm.2025.1703472 (PMC12745410; doi:10.3389/fcvm.2025.1703472)
Supplement: Supplementary Table 1 — Criteria for blockage grades using the CryoMST and corresponding fluoroscopy-guided pulmonary venography blockage grade assessment. [file Datasheet1.pdf]

Supplementary Table 1: Criteria for blockage grades using the CryoMST and corresponding fluoroscopy-guided pulmonary venography blockage grade assessment.

Level	Contrast agent	Saline
I occlusion	No contrast reflux	Balloon surface temperature lower than center temperature, maintained for ≥15s
II occlusion	Slight contrast reflux and minimal leakage	Balloon surface temperature lower than center temperature, maintained for 6 to 15s
III occlusion	Significant leakage and rapid contrast loss	Balloon surface temperature lower than center temperature, maintained for <6s
