# Supplementary material for: Evaluation of the novel multi-points surface thermometry cryoballoon in the treatment of paroxysmal atrial fibrillation
Source: Front Cardiovasc Med. 2025 Dec 15;12:1703472. doi: 10.3389/fcvm.2025.1703472 (PMC12745410; doi:10.3389/fcvm.2025.1703472)
Supplement: Supplementary Table 2 — Comparison of fluoroscopy time, fluoroscopy dose, and contrast agent usage between the first 8 patients and the second 8 patients in the CryoMST group. [file Datasheet2.pdf]

Supplementary Table 2: Comparison of fluoroscopy time, fluoroscopy dose, and contrast agent usage between the first 8 patients and the second 8 patients in the CryoMST group.

Variables	CryoMST Learning group (n=8)	CryoMST Post-learning group (n=8)	Diff (95%CI)	p
Fluoroscopy time (min)	16.53 ± 3.16	11.59 ± 1.21	4.94 (2.38, 7.51)	0.001
Fluoroscopy dose (mGy)	85.30 (71.88, 114.78)	77.00 (59.60, 95.50)	11.30 (-11.90, 39.00)	0.294
Contrast agent dosage (ml)	18.50 (14.75, 22.75)	11.50 (9.00, 20.25)	6.50 (-1.00, 13.00)	0.082

Data are presented as mean±SD or median (IQR).
